# A case of early pulmonary embolism following blunt trauma to chest and a long bone fracture: a case report

**DOI:** 10.1186/s41935-023-00327-4

**Published:** 2023-01-23

**Authors:** Yalini Thivaharan, Asurappulige Dayapala, Muthulingam Thanushan

**Affiliations:** 1 Colombo South Teaching Hospital, Kalubowila, Sri Lanka; 2 Department of Forensic Medicine, Faculty of Medicine, University of Sri Jayawardhanapura, Sri Jayawardhanapura, Sri Lanka

**Keywords:** Early pulmonary embolism, Deep vein thrombosis, Chest trauma, Long bone fracture, De novo thrombi

## Abstract

**Background:**

After substantial trauma, pulmonary embolism (PE) carries a high risk of morbidity and mortality. Early post-traumatic PE in the absence of deep vein thrombosis (DVT) is a distinct entity that might be connected to rare mechanisms or unidentified biochemical processes.

**Case presentation:**

A driver of a car was presented with worsening chest pain and shortness of breath (SOB) following a road traffic accident in which he suffered an impact on the chest against the steering wheel and a closed fracture of the right femur. Radiological investigations revealed a pulmonary embolism in the left posterior segment pulmonary artery, without evidence of internal chest injuries or DVT within 12 h from the incident. D-dimer, troponin I, and creatinine kinase were elevated without evidence of myocardial infarction or myocardial injury. Other parameters were within the normal range.

**Conclusions:**

Possibilities of early PE in the absence of detectable DVT could be due to hyper-coagulability states, clots from the lower extremity completely getting detached and embolizing to the pulmonary circulation, screening errors, and “de novo” thrombi in the pulmonary circulation. Chest trauma is an identified risk factor for early or late pulmonary embolism. Action of the post-traumatic adrenergic response causing vascular endothelial inflammation and the synthesis of circulating adhesion molecules leading to localized thrombosis have also been suggested as causes for this phenomenon. A greater understanding of rare risk factors for early PE and the possibility of rare complications of chest trauma is useful in detecting and treating them in time, reducing morbidity and mortality.

## Background

After substantial trauma, pulmonary embolism (PE), though relatively rare, carries a high risk of morbidity and mortality. According to reports, mortality rates with PE following injury range from 17% to 26% (Sing et al., 2006). PE typically develops following a deep vein thrombosis (DVT) of the lower limbs or pelvis and ultimately embolizes to the pulmonary circulation. Since this process takes time, studies have shown that PE usually occurs 5 to 7 days following trauma (Owings et al., 1997). But, according to a number of recent researches PE can occur within a few days of a trauma (Menaker et al., 2009). The incidence and timing of emergence of PE are still controversial (Coleman et al., 2015). Old age, long bone and lower extremity fractures, injuries to the head, pelvis, or spinal cord, prolonged immobility, and injury severity are considered as risk factors for PE following an injury (Paffrath et al., 2010).

However, early post-traumatic PE is rare and reported in very few instances in the medical literature (BennsM & Kim, 2014; Namdar Kazemi Darabadi et al., 2018). It is a distinct entity that might be connected to unidentified biochemical processes and might not even have originated in the peripheral veins (GC et al., 2009). Managing PE efficiently is a challenge to physicians, as they need to act quickly and strike a balance between risks and benefits of anticoagulation therapy.

This paper discusses a patient with radiologically diagnosed PE and two possible competing causes: long bone fracture and blunt chest trauma.

## Case presentation

A 41-year-old driver of a car was admitted to a primary care hospital, following a collision of his car with a lorry on the 22nd of July 2022. Due to a lack of facilities in the primary care hospital, he was immediately transferred to a tertiary care hospital for further management. On admission to the tertiary care hospital, he was found to be having an abrasion over the nasal bridge with underlying nasal bone fracture and abrasions over the right knee and a closed fracture of the shaft of the right femur only. There were no external injuries on the chest. But he complained of non-radiating, central chest pain with mild shortness of breath.

On admission, his vitals were unremarkable with the Glasgow coma scale of 15/15, equal air entry in both lungs, soft and non-tender abdomen. There was no tenderness on the chest region. Clinical signs of deep vein thrombosis (DVT) such as calf pain, leg swelling, and pain on passive movements of the legs have not been noted.

Detailed history revealed that during the collision, he had felt his chest hitting against the steering wheel of his car. His chest pain and shortness of breath worsened in the early hours of 23rd July 2022 (less than 12 h from the time of the accident), with no associated autonomic symptoms or demonstrable chest injury.

The patient had no history of recent prolonged immobilization, personal or family history of thrombotic events, or a history of COVID-19 infection. He had obtained the third dose of the COVID vaccine a couple of months ago. He is a non-smoker and used to drink alcohol occasionally.

His oxygen saturation was 97%. A chest radiograph showed unremarkable lung fields and did not reveal rib or sternal fractures or haemo-pneumothorax. The electrocardiogram (ECG) showed no dynamic changes, but his troponin I was elevated to 1.7ng/mL (reference limit less than 0.03ng/mL). An immediate medical referral was done to look for cardiac and non-cardiac causes for elevated Troponin I, such as myocardial infarction, pulmonary embolism, and cardiac contusions.

Transthoracic echocardiogram showed an ejection fraction of >60% without hypokinetic areas in the myocardium. The atria were not dilated.

**Fig. 1:**
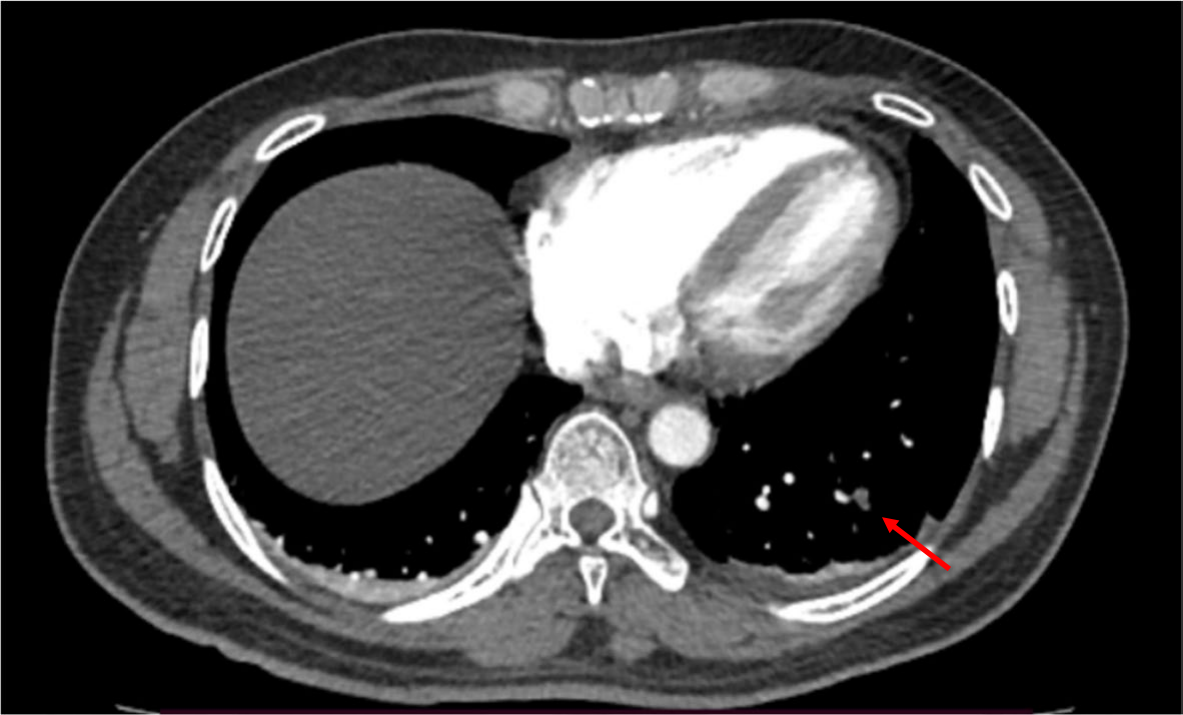
A CT pulmonary angiogram revealed a pulmonary embolism in the left posterior segment pulmonary artery

A CT pulmonary angiogram revealed a pulmonary embolism in the left posterior segment pulmonary artery (Fig. 1) with no evidence of right ventricular strain. The main pulmonary trunk and left and right main pulmonary arteries appeared normal with no filling defects. Venous duplex scan of lower extremities showed no evidence of DVT.

His D-dimer level was elevated at 3.40 mg/L (reference limit less than 1 mg/L). Full blood count was within normal limits. C-reactive protein peaked at 122 (normal range <5mg/L), fasting blood sugar was 122 mg/dL (normal range <100), creatinine kinase was elevated at 1826 (normal range <171), and liver enzymes were mildly elevated. The coagulation profile was normal.

## Discussion

A driver of a car was presented with worsening chest pain and shortness of breath following a road traffic accident. He was diagnosed to be having PE within 12 h from the injury both clinically and radiologically, without evidence of DVT.

Though chest pain and shortness of breath can be due to chest trauma, myocardial infarction, and PE in this case, clinical and radiological examination and ECG could exclude chest trauma and myocardial infarction with a reasonable degree of accuracy. Cardiac troponins are the most sensitive and the most specific biochemical marker indicating myocardial injury. But troponin can also get elevated in a few non-cardiac causes such as acute PE, sepsis, and acute renal failure. Elevation of Troponin in PE is attributed to the increased mechanical load on the right ventricle caused by the increased pulmonary vascular resistance due to pulmonary artery obstruction (Kilinc et al., 2012). In this patient, the rise in creatinine kinase, a compact enzyme that is found in tissues where energy demands are high, may be elevated due to either the muscle injury following femur fracture (Baird et al., 2012) or due to the PE having a deleterious effect on the cardiac muscle of the right ventricle (Adams III et al., 1992).

According to research, up to 30% of people with PE have them restricted to subsegmental or smaller arteries (Oser et al., 1996). In the absence of a central PE, the clinical relevance of peripheral emboli in subsegmental or smaller pulmonary arteries is yet unknown. Additionally, there is a debate over how these minor peripheral emboli should be treated and whether doing so will lead to better results (Novelline et al., 1978). In a study conducted by Menaker et al. (2009), majority of PE were located in the main, lobar and segmental arteries, and most of the emboli were clinically significant. There is no such thing as clinically minor PE (Handler & Feied, 1995).

While Menaker et al. (2009) and Brakenridge et al. concluded through studies that a PE can occur as early as 4 days from the onset of injury, Coleman et al. (2015) found in 2015 that 42.9% of patients with PE following trauma, was diagnosed within 72 h of hospitalization. According to the most recent study by Gelbard (2016) more than half of the early PEs occurred within 24 h of trauma. On the ground of these studies, we can suspect that an ‘early’ PE may be associated with a different pathophysiological mechanism. However, much more research is needed to come to a conclusion.

There can be several possibilities for early pulmonary embolism following trauma. One is an undiagnosed congenital or acquired prothrombotic conditions. Hypercoagulable state becomes more prominent in the first 4 days following trauma (Schreiber et al., 2005). Another could be, as Velmahos (2009) proposed, patients with early post-traumatic PE in the absence of DVT, could be due to pulmonary clots that are generated within the lungs. Furthermore, Brakenridge (2011) postulated that a long bone fracture alone is an independent risk factor of early PE. He stated that there could be an underlying molecular phenomenon linked to fractures causing early PE.

Furthermore, the literature survey reveals that fat embolism appears to be more commoner than thromboembolism following long bone fractures, with its incidence widely ranging from 47% to 100% distinguished by advanced diagnostic tools (Gurd & Wilson, 1974), whereas fat embolism has been diagnosed through autopsy examination in 68% to 82% (Mudd et al., 2000) who succumbed to major trauma. Accurate incidence of thrombembolism following long bone fractures remains unclear in the literature survey.

Bahloul et al. (2020) in a narrative review list four possibilities of early PE in the absence of DVT. They are (i) rapidly developing post-traumatic hypercoagulable disorders (ii) clots that form in the lower extremity veins completely embolizing to the pulmonary circulation without leaving any residues behind (iii) screening errors and undetected upper extremity DVTs and (iv) thrombi in the pulmonary circulation are “de novo” and not generated from the peripheries.

Chest trauma is also an identified risk factor for the development of early or late pulmonary embolism. Velmahos et. al (2009) theorized that in the absence of DVT, there can be other pathophysiology that resulted in PE. Knudson (2011) has reported a case of PE following a severe chest injury and suggested localized inflammation, occult vascular injury, and low flow state occurring following chest trauma as a likely aetiopathogenesis for “in situ” formation of thrombus in the pulmonary circulation. This is a probability in the case under discussion in the view of the history of chest impact though there were no demonstratable external or internal chest injuries.

Other risk factors for PE such as obesity, old age, sepsis, and acute renal failure (Paffrath et al., 2010) did not exist in our patient. The possibilities of congenital prothrombotic conditions were also minimal in this case.

Additionally, the development of pulmonary thrombosis may be influenced by the action of the post-traumatic adrenergic response, which causes vascular endothelial inflammation and the synthesis of circulating adhesion molecules, leading to localized thrombosis and rapid occlusion (Morris et al., 2007). More research is required on this topic.

## Conclusions

In trauma cases, treating doctors should have a high degree of suspicion of PE, especially when there is unexplained chest pain and dyspnea. We think that a greater understanding of rare risk factors for PE as well as the possibility of rare complications of chest trauma will be useful in detecting and treating them in time, reducing morbidity and mortality.

## Data Availability

No objections in sharing data.

## Abbreviations

PE: Pulmonary embolism
DVT: Deep vein thrombosis
ECG: Electrocardiogram
CT pulmonary angiogram: Computed tomography pulmonary angiogram.
